# Extrapyramidal side effects in first-episode schizophrenia treated with flupenthixol decanoate

**DOI:** 10.4102/sajpsychiatry.v27i0.1568

**Published:** 2021-01-11

**Authors:** Francois-Pierre Joubert, Bonginkosi Chiliza, Robin Emsley, Laila Asmal

**Affiliations:** 1 Department of Psychiatry, Faculty of Medicine and Health Sciences, Stellenbosch University, Cape Town, South Africa; 2 Department of Psychiatry, Nelson R Mandela School of Medicine, University of KwaZulu-Natal, Durban, South Africa

**Keywords:** flupenthixol, Parkinsonism, dystonia, akathisia, tardive dyskinesia

## Abstract

**Background:**

Concern for the development of extrapyramidal side effects (EPSEs) represents a barrier to the routine use of long-acting injectable (LAI) antipsychotic medication in patients with first-episode schizophrenia (FES). Flupenthixol decanoate is a first-generation antipsychotic, which is readily available in the public healthcare system in South Africa.

**Aim:**

The aim of this study was to describe the nature, occurrence and severity of EPSEs and their impact on patients with FES over 12 months of treatment with flupenthixol decanoate (fluanxol depot).

**Setting:**

The study was based in Cape Town, South Africa, and patients with FES were recruited from inpatient services at Stikland and Tygerberg Hospitals and surrounding psychiatric clinics. This was a sub-study of a larger study, which examined several outcomes in patients with FES treated with the lowest effective dose of flupenthixol decanoate.

**Methods:**

The Extrapyramidal Symptom Rating Scale (ESRS) was used to assess both subjective experience and objective measures of EPSEs in a cohort of patients with FES (*N* = 130). The relationship between demographic and clinical risk factors for individual subsets of EPSEs was also determined.

**Results:**

In the context of an overall good 12-month tolerability, EPSEs peaked at month 3. Patients with akathisia were more likely to have greater symptoms of depression, and Parkinsonism was predicted by higher Positive and Negative Syndrome Scale scores (independent of medication dosage). Black and white patients showed higher total ESRS and higher subjective ESRS scores, compared with patients of mixed ancestry, and white patients scored higher on Parkinsonism ratings.

**Conclusion:**

Flupenthixol decanoate is well tolerated in patients with FES. Certain clinical features of schizophrenia may be related to EPSEs. Ethnicity is a socio-cultural construct, and hence the differential risk of EPSEs should be interpreted according to ethnicity. Variations in the environment, diet, substance use and genetics may all affect the pharmacokinetics and pharmacodynamics of psychotropic drugs and warrant further investigation.

## Introduction

There is a growing interest in the use of long-acting injectable (LAI) antipsychotics in patients with first-episode schizophrenia (FES).^1,2^ These offer the advantage of explicit adherence combined with effectiveness and low costs, in the case of first-generation antipsychotic (FGA) depots.^1,2^ Flupenthixol decanoate is a first-generation LAI antipsychotic, which displays certain atypical qualities.^3^ It has a half-life of approximately 3 weeks, is highly protein bound (99%) and is mostly excreted by the liver.^4^ Like other LAI antipsychotics, flupenthixol decanoate is highly effective in treatment, but remains underutilised clinically in part because of the familiar problem of extrapyramidal side effects (EPSEs),^5^ which patients with FES are especially sensitive to.^6^

EPSEs contribute significantly to disability and cause social embarrassment. Akathisia, the subjective feeling of inner restlessness,^7^ has been associated with non-adherence, depression and suicide.^8^ Acute dystonia, an acute involuntary contraction of a muscle group, tends to be painful and distressing for patients and their families.^9,10^ Parkinsonism is potentially debilitating to the patient and can mimic negative symptoms, thus obscuring clinical features of schizophrenia.^10^ The tardive dyskinesias are late emerging hyperkinetic movements with numerous clinical manifestations, including chorea, athetosis and tardive akathisia.

Overall, there are few studies that examined EPSEs related to LAI antipsychotics in patients with FES.^2^ Further, EPSEs are frequently poorly reported, or are reported in ways that are not always helpful to clinicians.^11^ Given the concern that EPSEs might be a deterrent to the use of LAI antipsychotics in patients with FES (and FGAs in particular), the aim of this study was to describe the nature, occurrence and severity of EPSEs and their impact on patients with FES over 12 months of treatment with flupenthixol decanoate. We explored both the objective (clinician rated) and subjective (patient reported) experience of EPSEs, as well as the relationship between EPSEs and clinical symptoms of schizophrenia.

## Method

### Setting

This was a sub-study of a larger study, which aimed to examine the clinical, functional and biological outcomes in patients with FES treated with the lowest effective dose of flupenthixol decanoate medication over 12 months. The study was based in Cape Town, South Africa, and patients with FES were recruited from inpatient services at Stikland and Tygerberg Hospitals and surrounding psychiatric clinics between 2007 and 2011.

### Participants

Participants were both males and females aged between 16 and 45 years meeting the Diagnostic and Statistical Manual of Mental Diseases, Fourth Edition, Text Revisions (DSM-IV TR)^12^ criteria for a first episode of schizophreniform disorder, schizophrenia or schizoaffective, and for ease of reporting they are referred to as FES. Participants were excluded if they had a current substance use disorder, an intellectual disability, were previously treated with a depot antipsychotic or ever been treated with antipsychotics for more than 4 weeks.

### Intervention

Patients received oral flupenthixol (1–3 mg/day for 7 days), followed by flupenthixol decanoate injections every 2 weeks throughout the study period. The initial dose was 10 mg every 2 weeks, and increases of 10 mg every 6 weeks were permitted, if clinically indicated, to a maximum of 30 mg every 2 weeks IMI. Additional oral flupenthixol was permitted at the discretion of the investigator, as was lorazepam, anticholinergics, propranolol, antidepressants and medication for general medical conditions. No benzodiazepines, propranolol or anticholinergics were permitted in the 12 hours prior to assessments. Patients were followed up for a 12-month period, and there were nine scheduled visits: at baseline, 1, 2, 4 and 6 weeks and at 3, 6, 9 and 12 months.

### Data collection

Socio-demographic, anthropometric and clinical data were collected at baseline including age, gender, ethnicity, diagnoses, duration of untreated psychosis and history of illicit substance use. Patients were assessed on intake by using the Structured Clinical Interview for DSM-IV,^13^ psychopathology severity was assessed by using the Positive and Negative Syndrome Scale (PANSS)^14^ and we used factor-analysis-derived symptom domains for positive, negative, depression or anxiety, excitement or hostility and disorganised symptoms.^15^ Depressive symptoms were assessed by using the Calgary Depression Rating Scale for Schizophrenia (CDSS).^16^ We measured insight by means of the Birchwood Insight Scale (BIS),^17^ which assesses three dimensions of insight: symptom awareness, illness awareness and need for treatment, as well as providing a BIS total score. We administered the Clinical Global Impressions (CGI) Scale as an overall clinician-determined summary of severity of illness measure.^18^ We assessed for extrapyramidal symptoms using the Extrapyramidal Symptom Rating Scale (ESRS). The ESRS is a well-validated rating scale used to assess EPSEs. It consists of five sections: a patient questionnaire and four subsections based on clinical observation and examination. The patient questionnaire asks patients to rate their subjective experience of EPSEs in the preceding week. The four subsections correspond with the four EPSEs: Parkinsonism, akathisia, dyskinesia and dystonia. Additional adverse event (AE) reporting was performed. Investigators underwent training and inter-rater reliability (IRR) testing. The IRR was > 0.75 for all scales. ‘Dropouts’ refers to withdrawal from the study that was either patient initiated (e.g. did not return for follow-up appointments, chose alternate treatment options) or clinician initiated (e.g. non-response, persistent side effects).

### Statistical analysis

Baseline measures for categorical variables were summarised as counts and percentages. Changes in ESRS scores (total and individual items, including subjective scores) over the various time points were summarised as least square means assessed by using variance estimation, precision and comparison (VEPAC) and normalised by using CoxBox transformation. Time points were normalised by using type III decomposition. We performed linear mixed-effect regression models for the following ESRS subscales: subjective ESRS, Parkinsonism, akathisia, tardive dyskinesia and acute dystonia, examining clinical (total PANSS, total depression, insight subscales) and demographic (age, gender and ethnicity) variables as potential predictors. We employed a mixed-effect linear regression model for repeated measures to evaluate a number of variables (age, ethnicity, gender, total PANSS score, total CDSS score, BIS subscale scores and flupenthixol dose) as predictors for EPSEs as measured by ESRS scores (total, subjective and subsections). Statistica (version 13.3) was used for all analyses and the level of statistical significance was set at *p* < 0.05.

### Ethical consideration

We obtained ethics approval from the Human Health Research Ethics Committee (HREC) of Stellenbosch University (Ref. N06/08/148). All participants provided written informed consent or assented, and if they were under age 18 years a parent or legal guardian provided consent.

## Results

### Demographic and baseline clinical details

Patients with FES (*n* = 130) were young (mean age 24.07 years), predominantly mixed-race ancestry (78%) and male (74%). Absolute numbers of EPSE AEs over 12 months were: akathisia (*n* = 20, 15%), Parkinsonism (*n* = 17, 13%), dystonia (*n* = 10, 7%) and dyskinesia (*n* = 8, 6%) (Table 1).

**TABLE 1:** Demographics and clinical features of participants with first-episode schizophrenia (*N* = 130).

Variable	Outcome			
	Mean	SD	*n*	%
Age in years	24.07	6.59	-	-
**Ethnic group**				
Mixed race	-	-	98	78
Black	-	-	18	14
White	-	-	10	8
Male:female	-	-	-	74:26
Highest school grade	10	2	-	-
Endpoint flupenthixol dose, mg/every 2 weeks	13	6.3	-	-
**EPSE count**				
Parkinsonism	-	-	17	13
Akathisia	-	-	20	15
Dystonia	-	-	10	7
Dyskinesia	-	-	8	6
**Baseline clinical scores**				
PANSS total score	94.78	16.48	-	-
CDSS total score	3.37	4.07	-	-
BIS total score	5.97	2.07	-	-
**Endpoint scores**				
PANSS total score	45.18	11.08	-	-
CDSS total score	0.94	2.48	-	-
BIS total score	6.25	1.90	-	-

SD, standard deviation; EPSE, extrapyramidal side effect; PANSS, Positive and Negative Syndrome Scale; CDSS, Calgary Depression Rating Scale for Schizophrenia; BIS, Birchwood Insight Scale.

### Total extrapyramidal symptoms scores over 12 months

**FIGURE 1:**
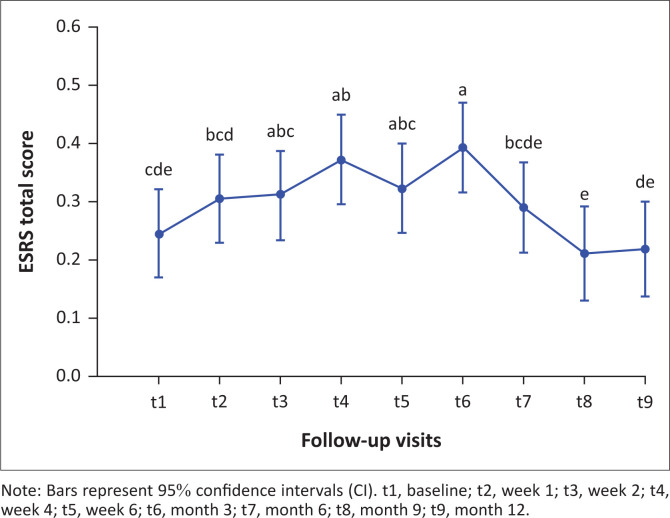
Total Extrapyramidal Symptom Rating Scale scores over 12 months of follow-up, displayed as least square means.

**FIGURE 2:**
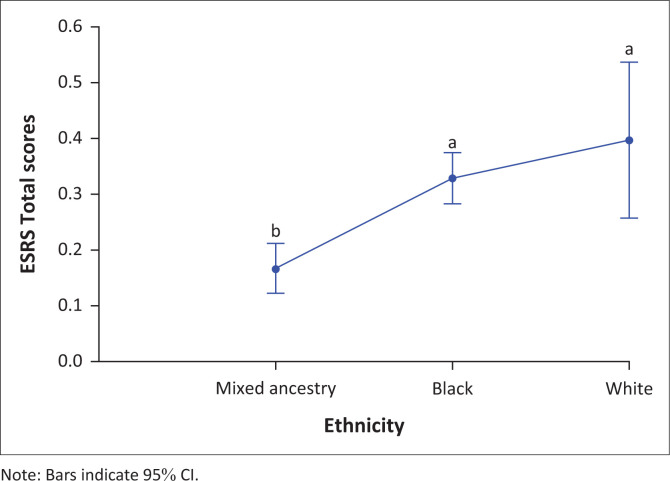
Total Extrapyramidal Symptom Rating Scale scores by ethnicity.

Total ESRS scores were relatively low over 12 months (Figure 1). Total ESRS scores peaked in the third month of treatment (Figure 1), which was associated with a significant number of dropouts (*p* = 0.03) (Supplementary Table 2). Total ESRS scores then declined approximately to baseline levels by 12 months. Specifically, ESRS scores were significantly lower at baseline compared with week 4 (*p* < 0.01) and month 3 (*p* < 0.01). Total ESRS scores were also lower at month 9 compared with week 2 (*p* = 0.04), week 4 (*p* = 0.03), week 6 (*p* = 0.01) and month 3 (*p* < 0.001). At 12 months, total ESRS scores were lower than at week 4 (*p* = 0.04), week 6 (*p* = 0.02) and month 3 (*p* ≤ 0.0001). Further, ESRS total score was significantly higher across the 12-month period overall for black (*p* ≤ 0.001) and white (*p* = 0.02) patients compared with patients of mixed ancestry. This was not significant for any specific time points (Figure 2).

**TABLE 2:** Linear mixed-effect repeated measure model: Clinical and demographic predictors of extrapyramidal side effects.

Measure	Coef.	95% CI	Std. Err.	Z	*P* > *z*
**Subjective EPSEs**					
PANSS total	-0.002	-0.01 – -0.003	0.01	-2.19	0.03*
CDSS total	0.03	0.00–0.07	0.02	1.96	0.05
**Parkinsonism**					
Ethnicity: Caucasian	1.06	0.18–1.94	0.45	2.35	0.02*
PANSS total	0.01	0.00–0.02	< 0.001*	2.02	0.04*
**Dystonia**					
Dose	0.21	0.13–0.28	0.04	5.48	< 0.001*
**Dyskinesia: nil predictors**					
**Akathisia**					
BIS: symptom attribution	0.02	0.00–0.05	0.01	2.11	0.04*

EPSE, extrapyramidal side effect; PANSS, Positive and Negative Syndrome Scale; CDSS, Calgary Depression Rating Scale for Schizophrenia; BIS, Birchwood Insight Scale.

* , Significant at *p* < 0.05.

### Subjective Extrapyramidal Symptom Rating Scale scores over 12 months

**FIGURE 3:**
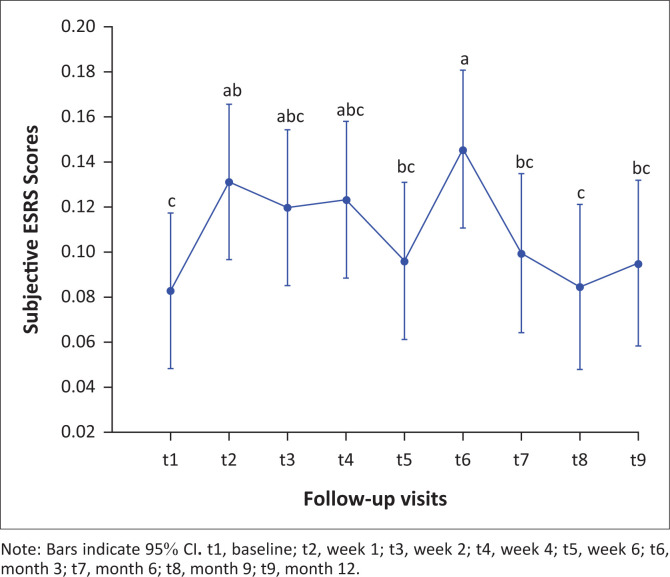
Subjective Extrapyramidal Symptom Rating Scale scores least square means over 12-month follow-up.

Subjective ESRS scores were relatively low over the 12-month study duration (Figure 3). The scores of the ESRS subjective component were significantly higher at week 1 compared with baseline (*p* = 0.02), that is, in the lead up to receiving the first depot antipsychotic, whilst receiving oral flupenthixol. Subjective ESRS symptoms were also significantly greater at month 3 compared with baseline (*p* < 0.001), and at month 9 compared with week 1 (*p* = 0.04). Thereafter, the subjective experience of ESRS symptoms was significantly lower when compared with month 3 at the subsequent three follow-up visits: at month 6 (*p* = 0.03), month 9 (*p* = 0.01) and month 12 (*p* = 0.02). White and black patients also reported experiencing significantly more EPSEs (*p* < 0.001) than patients of mixed ancestry.

### Relationship between extrapyramidal side effects and clinical and demographic factors

Table 2 summarises the statistically significant findings of the linear mixed-effect repeated measures model describing the relationship between EPSEs and clinical and demographic characteristics (refer to Appendix 1, Table 1-A1 for the full results of the model). Higher subjective ESRS scores were predicted by higher depression scores (*p* = 0.05) and lower total PANSS scores (*p* = 0.03). For the ESRS subscales, a higher Parkinsonism score was predicted by a higher total PANSS score (*p* = 0.043) and ethnicity, with white patients scoring significantly higher (*p* = 0.027). Acute dystonia was predicted by dose (*p* < 0.0001). Akathisia was predicted by a lower total PANSS score (*p* = 0.04) and higher scores for the symptom attribution subscale of the BIS (*p* = 0.028). No clinical or demographic variables emerged as predictors of tardive dyskinesia.

## Discussion and conclusion

In this study, we examined the subjective and objective nature of EPSEs in a cohort of patients with FES treated with flupenthixol decanoate, an LAI antipsychotic, over 12 months. Our study has two main findings. Firstly, we found that in the context of overall good 12-month tolerability, EPSEs peaked at month 3 and this peak was associated with a significant increase in dropouts. Secondly, we found evidence of an association between clinical symptomatology and subjective and total EPSEs.

Both subjective and total ESRS scores showed a similar trend, a statistically significant initial rise in scores followed by a decline to approximately baseline by the endpoint. This finding is in keeping with the onset of acute extrapyramidal symptoms in other studies. Dystonia is a particularly acute EPSE, and in 95% of cases the onset is within 5 days of antipsychotic initiation or within the few days after LAI antipsychotics are administered.^19^ The onset of Parkinsonism, on the other hand, occurs within days to weeks after antipsychotic administration with 50% – 75% occurring within the first month of treatment and 90% by 3 months.^20^ Similarly, the onset of akathisia is within the first month of antipsychotic administration in 50% of cases and within 3 months in 90% of cases.^20^

Schizophrenia itself may be associated with abnormal movements,^21^ and this may have contributed to the subjective and objective EPSEs that were present at baseline in our study. Abnormal motor findings were frequently recorded in the classical schizophrenia literature,^22^ with one study describing signs of abnormal movements in 66% of antipsychotic naïve patients with schizophrenia spectrum illness.^23^ A meta-analysis showed an odds ratio of 5.32 for spontaneous Parkinsonism in treatment naïve patients with schizophrenia.^24^ It has also been demonstrated that spontaneous Parkinsonism predicts medication-induced Parkinsonism^25^ and that motor features of schizophrenia may be ameliorated by antipsychotic treatment.^26^

Although ESRS totals and subjective ESRS scores followed a similar longitudinal course in our study, the onset of side effects differed. The subjective experience scores rose sharply, showing a statistically significant increase in the first week of the study, whilst on low-dose oral flupenthixol (1 mg daily) but prior to the initiation of depot antipsychotics. One possibility for this finding is that patients were aware of extrapyramidal symptoms before the signs were clinically apparent. This might be explained by the cognitive effects of neuroleptics (Awad, 1993; Lindstrom, 1994) in that dysphoria caused by neuroleptics may predict the onset of EPSEs.^29^ It has also been suggested that rating scales of EPSEs might not be sensitive enough to distinguish dysphoria caused by neuroleptics from similar syndromes such as depressive symptoms or EPSEs.^30^ Interestingly, we found that higher subjective ESRS scores were associated with higher CDSS (depressive) scores which may be a reflection of neuroleptic dysphoria. The period between the rise in objective experience scores and the rise in clinician-rated scores may offer a time frame during which interventions could reduce the eventual side effect burden and potentially improve treatment adherence. We also found that patients with higher subjective ESRS scores had lower PANSS scores. Although the reasons for this are not clear, one hypothesis is that as psychopathology improves, awareness of side effects also improves – a very ill patient could be too ill to be aware of or to complain of side effects. This might be supported by the fact that clinician-rated total ESRS scores were not influenced by PANSS scores.

Our second key finding was the relationship between clinical and demographic factors and individual extrapyramidal symptom subtypes, namely Parkinsonism, dystonia and akathisia. Patients with akathisia were more likely to be have greater symptoms of depression (as measured by the CDSS). In the International Suicide Prevention Trial (InterSePT), greater rates of akathisia were associated with higher PANSS anxiety or depression factor scores,^31^ which may be in keeping with our finding of higher depressive symptoms as measured by the CDSS in patients with akathisia. A number of risk factors for akathisia have been described, including treatment risk factors (higher dose, rapid increases in dosage and higher potency antipsychotics) as well as individual risk factors (higher age, female sex, greater overall psychopathology, negative symptoms, iron deficiency, cognitive dysfunction and mood disorder).^22^ However, these risk factors have been identified from a limited number of studies, and the validity of the individual risk factors, in particular, requires further investigation.

Parkinsonism was predicted by higher PANSS scores and ethnicity with white patients having higher rates. Parkinsonism has been associated with higher PANSS scores in previous studies.^31^ There is evidence that although dopamine blockade happens within hours of antipsychotic medication, Parkinsonism may be delayed by days or even weeks.^19^ In our study, we did not identify a dose–response relationship with Parkinsonism, which may be because in patients treated with LAI antipsychotics Parkinsonism can persist for weeks and even months despite dose reduction. Well-established risk factors of Parkinsonism include higher dose medication, addition of a second antipsychotic, older age, female sex, brain structural abnormalities and pre-existing Parkinsonism.^19^ Acute dystonia was associated with higher dosages, a well-established association,^32^ but we did not identify any other established risk factors for the condition such as younger age, stimulant use, mood disorders and ethnicity. This may be related to the relatively low incidence of dystonia over 12 months, which makes identification of multiple risk factors difficult. We were unable to identify risk factors associated with tardive dyskinesia during the 12-month follow-up. Tardive dyskinesias are typically seen after prolonged antipsychotic use, and it may be that a year of treatment might have been too short a time frame to evaluate for risk factors associated with the development of tardive dyskinesias.^33^

We found that ethnicity was a significant predictor for a number of outcomes. Black and white patients showed higher total ESRS and higher subjective ESRS scores, compared with patients of mixed ancestry. White patients also scored higher on Parkinsonism ratings. It has been hypothesised that ethnic differences in response to medications in general are largely mediated by pharmacokinetics, specifically gut absorption and first-pass metabolism, high levels of protein binding and hepatic elimination.^34^ This may be relevant in relation to flupenthixol decanoate, given that the drug is 99% protein bound and is eliminated primarily by the liver.^4^ Although genetically determined pharmacokinetic and pharmacodynamic differences may exist between ethnic groups, all populations irrespective of racial group exhibit substantial intra-population genetic variability.^35^ Ethnicity is a socio-cultural construct and may be an important proxy for other factors.^36^ These factors include variations in nutrition, dietary habits, weight, substance use (including tobacco, caffeine, alcohol and illicit substances), levels of air pollution, use of herbal remedies or over-the-counter medication, which may all affect the pharmacokinetics and pharmacodynamics of psychotropic drugs and may differ considerably between ethnic groups.^37,38^ Differences in help-seeking behaviour, societal attitudes and cultural interpretations of symptoms may also influence subjective experiences of side effects.^36^

The key strengths of this study were that it investigated a cohort of patients with FES who were well characterised with regular standardised assessments over a 12-month period. Patients had minimal exposure to antipsychotics at the start of the study, and approximately half were neuroleptic naïve following which all patients were treated with the same medication type where antipsychotic administration was assured. Limitations include the relatively small sample size for examining individual EPSE components. Further, we did not have a comparison group (such as oral antipsychotics) to compare emergent EPSEs in our population too. Finally, because of data limitations, we are unable to report on reasons for dropouts at each time point.

In conclusion, we found a favourable 12-month tolerability of flupenthixol decanoate, a first-generation LAI antipsychotics, in patients with FES. The medication warrants further investigation into its suitability as a first-line agent for patients with FES. Subjective EPSEs increased prior to objective findings, which may provide an opportunity for earlier intervention. Clinical and demographic variables were identified as risk factors for Parkinsonism, dystonia and akathisia. This requires further investigation, and in the case of ethnicity genetic vulnerability and environmental factors in individual populations should be prioritised.

## Appendix 1

**Table 1-A1:** Linear mixed effect repeated measures model describing the relationship between EPSEs and clinical and demographic characteristics.

Measure	Coef.	Std Err	*z*	*P*>*z*	95% CI
**Subjective EPSEs**					
Age	0.01	0.01	0.75	0.45	−0.01–0.03
Ethnicity	0.05	0.12	0.46	0.65	−0.18–0.28
Gender	0.25	0.14	1.85	0.07	−0.02–0.52
PANSS total	0.00	0.00	−2.19	0.03*	−0.01–0.00
CDSS total	0.03	0.02	1.96	0.05	0.00–0.07
BIS: symptom attribution	0.01	0.05	0.20	0.84	−0.09–0.11
BIS: symptom awareness	−0.02	0.05	−0.36	0.72	−0.11–0.07
BIS: need for treatment	0.08	0.05	1.45	0.15	−0.03–0.18
Dose	0.14	0.08	1.70	0.08	-.02–0.29
**Parkinsonism**					
Age	0.00	0.02	0.05	0.96	−0.03–0.04
Ethnicity	0.49	0.22	2.21	0.03*	0.06–0.92
Gender	0.38	0.26	1.48	0.14	−0.12–0.89
PANSS total	0.01	0.00	2.02	0.04*	0.00–0.02
CDSS total	0.02	0.04	0.65	0.51	−0.05 0.09
BIS: symptom attribution	−0.12	0.10	−1.15	0.25	−0.33–0.08
BIS: symptom awareness	−0.15	0.09	−1.71	0.09	−0.33–0.02
BIS: need for treatment	0.10	0.11	0.87	0.38	−0.12–0.31
Dose	0.16	0.16	1.02	0.31	−0.15–0.48
**Dystonia**					
Age	0.00	0.00	0.55	0.58	−0.01–0.01
Ethnicity	0.01	0.05	0.11	0.92	−0.10–0.11
Gender	−0.01	0.06	−0.14	0.89	−0.13–0.11
PANSS total	0.00	0.00	−0.09	0.93	0.00–0.00
CDSS total	0.00	0.01	−0.51	0.61	−0.02–0.01
BIS: symptom attribution	0.04	0.03	1.61	0.11	−0.01–0.09
BIS: symptom awareness	0.01	0.02	0.69	0.49	−0.03–0.06
BIS: need for treatment	−0.03	0.03	−1.34	0.18	−0.09–0.02
Dose	0.21	0.04	5.48	< 0.001*	0.13–0.28
**Dyskinesia**					
Age	0.00	0.00	−0.24	0.81	−0.01–0.01
Ethnicity	0.02	0.06	0.35	0.72	−0.10–0.14
Gender	−0.10	0.07	−1.45	0.15	−0.24–0.04
PANSS total	0.00	0.00	−0.14	0.89	0.00–0.00
CDSS total	0.01	0.01	1.12	0.26	−0.01–0.03
BIS: symptom attribution	−0.01	0.03	−0.50	0.62	−0.07–0.04
BIS: symptom awareness	−0.03	0.02	−1.30	0.19	−0.08–0.02
BIS: need for treatment	0.00	0.03	−0.10	0.92	−0.06–0.06
Dose	0.00	0.04	0.01	0.99	−0.09–0.09
**Akathisia**					
Age	0.00	0.00	−0.08	0.94	0.00–0.00
Ethnicity	−0.01	0.02	−0.29	0.77	−0.05–0.04
Gender	−0.01	0.03	−0.40	0.69	−0.07–0.04
PANSS total	0.00	0.00	−0.75	0.45	0.00–0.00
CDSS total	0.00	0.00	0.71	0.48	0.00–0.01
BIS: symptom attribution	0.02	0.01	2.11	0.04*	0.00–0.05
BIS: symptom awareness	0.00	0.01	−0.12	0.91	−0.02–0.02
BIS: need for treatment	0.00	0.01	0.39	0.70	−0.02–0.03
Dose	−0.01	0.02	−0.85	0.39	−0.05–0.07

EPSE, extrapyramidal side effect; PANSS, Positive and Negative Syndrome Scale; CDSS, Calgary Depression Rating Scale for Schizophrenia; BIS, Birchwood Insight Scale.

* , Indicates statistical significance at *p* < 0.05.
