# Erratum: Extrapyramidal side effects in first-episode schizophrenia treated with flupenthixol decanoate

**DOI:** 10.4102/sajpsychiatry.v27i0.1780

**Published:** 2021-12-06

**Authors:** Francois-Pierre Joubert, Bonginkosi Chiliza, Robin Emsley, Laila Asmal

**Affiliations:** 1 Department of Psychiatry, Faculty of Medicine and Health Sciences, Stellenbosch University, Cape Town, South Africa; 2 Department of Psychiatry, Nelson R Mandela School of Medicine, University of KwaZulu-Natal, Durban, South Africa

In the version of the article initially published, Joubert F-P, Chiliza B, Emsley R, Asmal L. Extrapyramidal side effects in first-episode schizophrenia treated with flupenthixol decanoate. S Afr J Psychiatr. 2021;27(0), a1568. https://doi.org/10.4102/sajpsychiatry.v27i0.1568, a sentence in the ‘Conclusion’ section of the abstract was given incorrectly. The correct sentence should be ‘Ethnicity is a socio-cultural construct and the differential risk of EPSEs according to ethnicity should be interpreted accordingly’ instead of ‘Ethnicity is a socio-cultural construct, and hence the differential risk of EPSEs should be interpreted according to ethnicity’.

This correction does not alter the study’s findings of significance or overall interpretation of the study’s results. The publisher apologises for any inconvenience caused.

